# The Immune-Modulatory Role of Apolipoprotein E with Emphasis on Multiple Sclerosis and Experimental Autoimmune Encephalomyelitis

**DOI:** 10.1155/2010/186813

**Published:** 2010-05-31

**Authors:** Hong-Liang Zhang, Jiang Wu, Jie Zhu

**Affiliations:** ^1^Department of Neurology, The First Hospital of Jilin University, Changchun 130021, China; ^2^Department of Neurobiology, Care Sciences, and Society, Karolinska Institute, Stockholm 141 86, Sweden

## Abstract

Apolipoprotein E (apoE) is a 34.2 kDa glycoprotein characterized by its wide tissue distribution and multiple functions. The nonlipid-related properties of apoE include modulating inflammation and oxidation, suppressing T cell proliferation, regulating macrophage functions, and facilitating lipid antigen presentation by CD1 molecules to natural killer T (NKT) cells, and so forth. Increasing studies have revealed that APOE *ε* allele might be associated with multiple sclerosis (MS), although evidence is still not sufficient enough. In this review, we summarized the current progress of the immunomodulatory functions of apoE, with special focus on the association of APOE *ε* allele with the clinical features of MS and of its animal model experimental autoimmune encephalomyelitis (EAE).

## 1. Introduction

**Figure 1:**
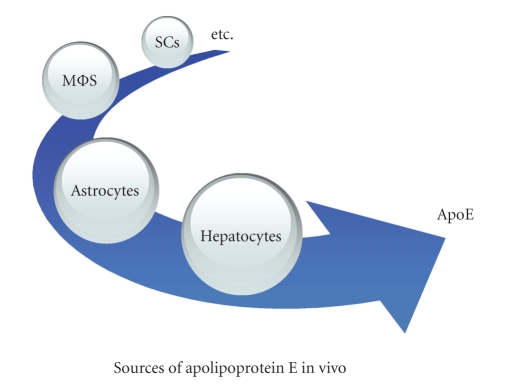
The synthesis and secretion of apoE are found in spleen, brain, lung, kidney, peripheral nerves, muscular tissue, adrenal, ovary and testis, and so forth. Hepatic parenchymal cells are the principal apoE-producing cells in mammalians, presumably accounting for 60% to 75% of plasma apoE, followed by astrocytes, which are the main apoE-producing cells in the brain, macrophages (MΦs), and nonmyelinating Schwann cells (SCs), and so forth.

Apolipoprotein E (apoE) is a 34.2 kDa glycosylated protein with 299 amino acid residues. The gene encoding apoE, APOE *ε*, lies on the 19th chromosome. There are three isoforms of apoE in human, namely, apoE2, apoE3, and apoE4 [1] (Table 1). However, murine apoE has only one isotype, which resembles human apoE3 in terms of lipoprotein-binding preferences [2, 3]. Hepatic parenchymal cells are the principal apoE producing cells in mammalians, presumably accounting for 60% to 75% of plasma apoE [4] (Figure 1). Its synthesis and secretion have also been found in the spleen, brain, lung, kidney, and so forth. In the nervous system, apoE mRNA is present in astrocytes, nonmyelinating Schwann cells, ependymal cells, neurons, and so forth [5, 6]. ApoE has been widely studied in cholesterol transport, atherosclerosis and cardiovascular diseases [7, 8], neurodegenerative diseases such as Alzheimer's disease [9, 10], and mild cognitive impairment [11, 12]. In addition, apoE functions on the immune system by suppressing T cell proliferation [13] and neutrophil activation [14], regulating macrophage functions [15–18], and facilitating the presentation of lipid antigen by CD1 molecules to natural killer T (NKT) cells, [19, 20] as well as modulating inflammation and oxidation [21]. The effects of apoE on immune responses have been shown to be extensive and some of them depend upon ligands, concentrations, and lipid-binding states. By these properties, apoE functions in both physiological and pathophysiological conditions at multiple levels. In this review, we outlined the immuno-modulatory properties of apoE, with special focus on the association of APOE *ε* allele with the clinical features of multiple sclerosis (MS) and of its animal model experimental autoimmune encephalomyelitis (EAE).

## 2. The Role of ApoE in Innate and Adaptive Immunities

The nonlipid-related properties of apoE were originally discovered as an inhibitory effect of plasma lipoproteins on T cell proliferation in vitro [22–24]. Succedent investigation ascribed this action to apoE, with a series of studies revealing that both apoE-containing lipoproteins and synthetic apoE peptides could inhibit antigen—and mitogen-stimulated T lymphocyte proliferation by downregulating DNA synthesis and by reducing phospholipid turnover in T cells [25–27], as well as modifying the function of interleukin(IL-2) receptor or modifying intracellular signaling pathways [28, 29]. Furthermore, apoE can downregulate the T helper (Th 1) 1 cell-mediated immune response [30, 31]. It is noteworthy that a peptide containing the receptor-binding region (residues 133–149), COG133 other than apoE holoprotein, is enough to suppress the response [32]. 

ApoE can neutralize lipopolysaccharide (LPS), attenuate the inflammatory responses, and, thus, reduce LPS-induced lethality [33]. ApoE deficiency results in impaired immune responses in mice to Listeria monocytogenes [34, 35], as well as to tuberculosis [36]. Ophir et al. showed that injection of LPS led to significantly higher production of pro-inflammatory cytokines such as IL-1*β*, IL-6, and tumor necrosis factor-*α* (TNF-*α*) in human APOE *ε*4 transgenic (Tg) mice than in APOE *ε*3 Tg ones [21]. 

Activation of macrophages is crucial in the initiation and effector phases of both the innate and adaptive immunities [37, 38]. Macrophages from apoE-deficient mice stimulated by exogenous antigen are more effective in upregulating the expression of pro-inflammatory cytokines, main histocompatibility complex (MHC) class II molecules, and costimulatory molecules in vitro [39]. Moreover, apoE suppresses the production of pro-inflammatory cytokines such as TNF-*α* and IL-1*β* by macrophages in an isoform-specific manner (E2 > E3 > E4) [40]. Conversely, classical activation of macrophages by inflammatory stimuli such as LPS, interferon(IFN-*γ*), TNF-*α*, and IL-1*β* was simultaneously accompanied with downregulation of apoE production [41, 42]. ApoE suppressing the inflammatory signaling in macrophages, and vice versa, indicate an intricate apoE-mediated feedback regulation of inflammatory responses. The production of nitric oxide (NO) represents one of the principle features of activated macrophages. Treatment of macrophages and microglia with apoE increases NO production [43]. After inflammatory stimulation, macrophages of APOE *ε*4 Tg mice produce a higher level of NO than of APOE *ε*3 Tg mice, which is coupled with an increased arginine uptake, depending on p38 mitogen-activated protein kinase (MAPK) [40, 44]. These findings point to the immuno-regulatory dysfunction in APOE *ε*4 carriers. Similar to apoE-deficient mice, APOE *ε*4 Tg mice seem to bear an insufficiency to deal with inflammatory insults.

**Figure 2:**
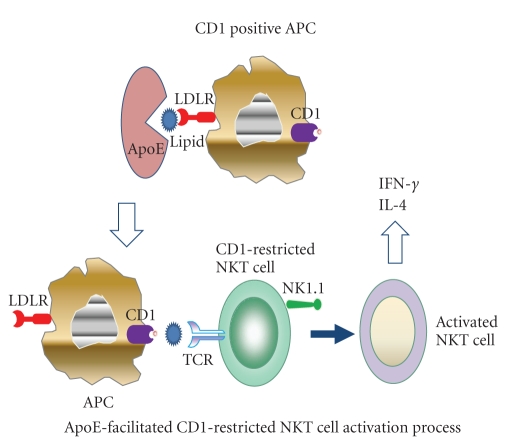
ApoE facilitates the activation of CD1-restricted natural killer T (NKT) cells. Inactivated NKT cells express surface marker of NK1.1 and a semiinvariant T cell receptor (TCR). ApoE facilitates lipid antigen-presentation by CD1 positive antigen presenting cells (APCs) mainly through binding to low-density lipoprotein receptor (LDLR). CD1 molecules present lipid antigens to NKT cells. Upon ligation to CD1 via TCR, naive NKT cells are activated, and respond rapidly to secrete high levels of IFN-*γ* and IL-4, which play a critical role in the modulation of immune and inflammatory responses.

APOE-knockout mice bear abnormal humoral and cellular immune responses [45]. Although the antigen-presenting function of macrophages seems to increase in apoE-deficient mice [39], there still lacks evidence that apoE directly functions on the antigen presentation process. This increase might be due to the enhanced susceptibility to inflammatory stimulation in apoE-deficient mice, resulting in higher expression of MHC class II molecules and costimulatory molecules on macrophages, or due to the elevated tendency to the Th1 immune response [31]. However, the role of apoE in facilitating lipid antigen presentation by CD1 molecules to NKT cells has been extrapolated to be of great importance in autoimmune diseases [46–49]. CD1 molecules (CD1a-d in humans and CD1d in mice), similar in structure to the MHC class I molecules, resemble MHC class II molecules in function in that they can present lipid antigens to NKT cells, in which process apoE is implicated. Upon binding to CD1 via TCR, naive NKT cells respond rapidly to secrete high amounts of IFN-*γ* and IL-4, which play a critical role in the regulation of immune responses [49] (Figure 2). More recently, B cells have been demonstrated to utilize the apoE-mediated pathways of lipid antigen presentation more efficiently than dendritic cells [50]. In terms of CD1-mediated self-lipid presentation, apoE might be involved in autoimmune diseases like MS via facilitation of self-lipid antigen presentation to NKT cells [51–53]. 

The exact mechanisms by which apoE isoforms affect the immunity remain unclear. However apoE has been presumed to influence multiple signaling pathways. ApoE isoforms might be in part responsible for the differential modulation of the redox-sensitive transcription pathways such as nuclear factor-*κ*B (NF-*κ*B) and MAPK [21, 54, 55]. Alternatively, apoE can act by binding to different cell surface receptors [56] to exert different functions, among which LDLR-related protein (LRP) is postulated to mediate the nonlipid-related effects of apoE [57, 58].

## 3. The Role of ApoE in MS

MS is primarily a chronic inflammatory demyelinating disorder in the central nervous system (CNS) characterized by focal infiltration of lymphocytes and macrophages, and subsequent immune-mediated damage to myelin and axons [59]. Aetiology of MS is suggested to be multifactorial and pathogenesis of it is still underclarified. A variety of studies have focused on the isoform-dependent role of apoE in MS, with an exclusive aim at finding an association of APOE *ε* allele with MS, just as the well-established one of APOE *ε*4 with Alzheimer's disease [60]. A general hypothesis is to assume that apoE genotypes can influence mechanisms of maintenance and repairing of the nervous system, which leads to distinct clinical courses in relation to the presence of a certain allele. A study on MS with magnetic resonance imaging (MRI) revealed lower total brain volumes in *ε*4 allele carriers as compared with non-*ε*4-carriers [61]. This finding provides new evidence that links APOE-*ε*4-related impaired restoration with severe tissue destruction in MS. However, considering the relatively low gene frequency of APOE *ε*4, especially APOE4/4 genotype in normal populations, the most probably implicated genotype affecting MS, it remains far from drawing a conclusion before more larger population-based studies are conducted.

### 3.1. ApoE As a Biological Marker in MS

Presently the useful biomarkers to assess the course and prognosis of MS are lacking. The ideal biomarker is one that reflects the neuropathology of MS lesions and, thus, has very high specificity and sensitivity, and that has a simple reproducible testing technique. Many studies reported the markers of inflammatory and immunological processes in MS [64]. In several studies aiming at finding specific biomarkers in the cerebrospinal fluid (CSF) of human Guillain-Barré syndrome (GBS), an analog of MS in the peripheral nervous system, apoE was shown to decrease in CSF in GBS patients by proteomic analysis and ELISA [65, 66]. Similarly, the levels of apoE in CSF decreased in MS [67, 68]. Furthermore, a study showed lower apoE concentrations in the serum of MS patients than in healthy subjects [69]. As there is limited permeability of the blood-brain barrier (BBB) to apoE [70, 71], this decrease in CSF might result from a reduction of local apoE synthesis and secretion by brain tissue, as a part of the suppressed systemic production of apoE in acute phage reaction, while it is difficult to explain the decreased apoE concentrations in serum [3]. However, other studies failed to show such a decrease of apoE in CSF [69, 72], or even presented completely opposite results in MS patients [73]. No correlation of apoE in serum or CSF with age, clinical course, or prognosis was found [68]. Considering the distinct status of in vivo inflammation and BBB integrity in different stages of MS, these conflicting results seem educible before larger investigations with more detailed stratification are conducted.

### 3.2. APOE *ε* Allele and Clinical Features of MS

The involvement of apoE in MS, although far from being elucidated, has been indicated by identification of the 19q13 chromosome as a candidate gene for autoimmune diseases from linkage analysis [74, 75], such as systemic lupus erythematosus [76]. APOE *ε* distribution in healthy populations is presented by two large-scale studies in Table 2. The gene frequencies of APOE *ε*2, 3 and 4 are 0.077, 0.773, and 0.150, respectively, as reported by Utermann et al. [62], and 0.078, 0.783, and 0.139 by Menzel et al. [63]. Regarding the geographical distribution of APOE *ε*4, there was a south-to-north gradient of APOE *ε*4 frequencies in Europe, with the frequency of APOE *ε*4 rising from only 10–15% in the south to 40%–50% in the north. And this is also the case in Japan [77]. The incidence of MS resembles APOE *ε*4 allele frequency in terms of geographical distribution [78]. Thus a hypothesis on this involvement is that a higher gene frequency of APOE *ε*4 is associated with MS. So far, most studies have not confirmed an alteration in APOE *ε* allele distribution in MS [79, 80]. The association between APOE *ε*4 homozygosity and MS was not investigated in most studies, since the APOE 4/4 homozygotes are rare both in healthy subjects and in MS patients [63, 81]. Only a limited number of studies attempted to but were unable to provide a positive conclusion [82, 83]. However, one exception is Høgh et al.'s study, in which homozygosity for APOE *ε*4 is more common in MS patients [84]. Anyhow, more large-scale studies are needed to elucidate the APOE *ε* allele distribution in MS.

Data from animal experiments suggested that BBB dysfunction resulting from apoE deficiency might lead to more susceptibility to EAE. Although there is no direct evidence that apoE contributes isoform dependently in maintaining BBB integrity, considering the preferences of apoE subtypes binding to lipoproteins and apoE receptors (Table 1), apoE isoforms may differ in protecting human from MS. Despite these presumptions, previous allelic association studies did not confirm the suggested relation of APOE *ε*4 allele with liability to MS [85–89]. A recent study reported that African Americans female MS patients who were APOE *ε*4 carriers had an earlier age of onset than Caucasian female MS patients, indicating that APOE *ε* allele might not be the independent factor to determine the age of onset in MS [90]. Albeit Chapman et al. found an earlier age of onset in the APOE *ε*4 carriers [91], most of others failed to find such positive associations [53, 68, 92–94].

**Figure 3:**
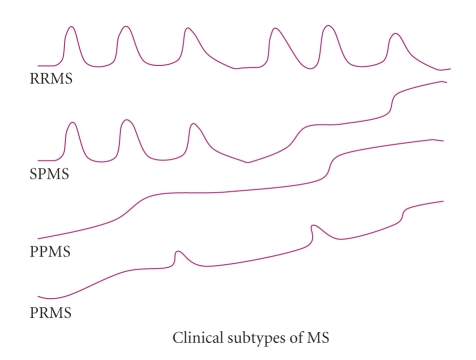
The four common clinical subtypes of MS are illustrated. RRMS (Relapsing-remitting MS). At least one clinical attack resulting from demyelination (relapsing phase) follows by complete or partial recovery (remitting phase), after the first recovery from an attack. SPMS (Secondary progressive MS). Symptoms are continuously and gradually worsening, after a period of RRMS. PPMS (Primary progressive MS). Symptoms keep worsening after the onset, without obvious relapsing-remitting phases. PRMS (Progressive relapsing MS). MS with characteristics of both PPMS and RRMS.

The classification of MS is based on the clinical observations, neuroimaging findings, and histopathological studies, as well as laboratory examinations. At least four clinical subtypes of MS have been identified, including relapsing-remitting MS (RRMS), which accounts for approximately 85% of all MS cases, primary progressive MS (PPMS), secondary progressive MS (SPMS), and progressive relapsing MS (PRMS). (The clinical course of MS subtypes is illustrated as in Figure 3.) Up to date, there has not been clear evidence for an association of apoE polymorphism with a specific clinical subtype of MS. Most studies denied the association between a particular APOE *ε* allele or genotype with MS subgroups, while the relatively small sample size limited the statistical power of the research [83, 95].

Currently, the most appropriate method to quantify severity and rate of progression is Expanded Disability Status Scale (EDSS), which is nonlinear, providing a mean value that represents mostly motor deficits. N-acetylaspartate-creatine ratio, as an index of axonal damage, is lower in the patients with MS and an *ε*4 allele, indicating that APOE *ε*4 allele correlated with MS disease severity [93]. An analysis of 614 patients with MS from 379 families indicated that APOE *ε*4 carriers were more likely to be involved in severe diseases [88]. A more recent study, on the other hand, suggested that the association of apoE polymorphisms with disease severity in MS was only in females [96]. Moreover, APOE *ε*4 was not associated with a more severe clinical course and did not appear to influence recovery of exacerbations, as some researchers revealed [97–100]. One neuroimaging study also showed a negative correlation between APOE *ε* allele and disease severity [101]. The conflicting results might be due to the heterogeneity of clinical manifestations in MS or due to the relatively small sample size in these studies. Another explanation is that the relation of APOE *ε*4 with clinical severity is probably not very strong [72].

Whether APOE *ε* allele influences the relapse or progression of MS is not clear. Relapse can be defined as a clinical attack resulting from demyelination, apart from the first episode of MS, characterized by three-phase clinical course, namely, onset, nadir, and recovery stages; the latter of which can either be complete or partial. It is assumed that a certain APOE *ε* allele is associated with more rapid relapse or progression. However, the results obtained from previous studies are contradictory. Most results proved the hypothesis of an association between the APOE *ε*4 allele and rapid progression of the disease [53, 84, 88, 91, 102], whereas others did not [79, 80, 103]. Neuroimaging data demonstrated accelerated brain-tissue loss and a higher proportion of lesions evolving into “black holes” in MS patients with APOE *ε*4. The annual reduction in brain volume in APOE *ε*4 carriers is five-fold higher than non-*ε*4-carriers [104]. There are also indications that the APOE *ε*4 allele is related to more severe progression of MS, as measured by MRI markers [105]. Some studies reported that the presence of the APOE *ε*4 allele was associated with a poorer outcome after evaluation with EDSS, progression index, and so forth [53, 88, 106], while a meta-analysis did not support this finding [80, 86, 95, 107–109]. It is worth noting that most of the aforementioned studies are retrospective cross-sectional ones, but not prospective longitudinal ones. The preexisting records, in such cases, were recorded for purposes other than validating the hypothesis and, therefore, might result in incomplete followups needed for the evaluation of progression or relapse in MS patients. Although still far from clarified, the apoE *ε*4 isoform has a higher affinity than the others to lipid molecules, and is thus postulated to associate with the poorer outcome in MS, which is related to a compromised capacity of remyelination or regeneration [95].

The recognition of the importance of cognitive impairment in MS patients is increasing. Cognitive impairment is now considered one of the earliest manifestations of the disease, which can affect up to 65% of MS cases [110, 111]. The association between APOE *ε*4 allele and cognitive deficits in MS has been verified by dozens of studies [112–114]. However, there usually lacks robust interpretation of the cognitive impairment in MS in most studies, which can either be only a symptom on account of myelin damage and axonal loss, suggesting the severity of the disease, or be at a subclinical stage of neurodegenerative diseases such as Alzheimer's disease, irrelevant of the disease of MS per se. Hence findings, either positive or negative, must be interpreted with caution [115]. Anyway, cognitive impairment in newly diagnosed MS is intriguing, since it may be of help to evaluate the disease. Besides, the depression is common in MS and is thought to interplay with cognitive and non-cognitive activities, but efforts to explain its aetiology, from effects of drugs, to physical disability, cognitive deficits, fatigue, and disease duration have not been successful. Recent studies have shown a relation between depression and demyelination in MS [116, 117]. Interestingly, the presence of the APOE *ε*2 allele seemed protective against depressive symptoms in MS [118], although further studies are needed to explain the mechanisms implicated.

## 4. The Role of ApoE in EAE

EAE, which is a CD4+ T cell-mediated experimental disorder in the CNS, has been proposed as an animal model for MS to investigate the pathogenesis and to test new therapeutic strategies. Since the first classical study by Rivers in monkeys immunized with brain homogenate, virtually all mammalian species have been revealed to be susceptible to EAE [119]. EAE has been in the last 30 years, the most frequently used animal model to study MS. Although restricted to the limited number of apoE-related EAE studies, current knowledge from literature points towards an affirmatory role of apoE in the pathogenesis of EAE. In as early as 1980s, activated macrophages and lesions in the CNS were proposed to be causally related to the increased apoE in plasma in EAE [120]. Exacerbated EAE in apoE-deficient mice was later demonstrated to be concomitant with significantly more infiltration of lymphocytes and macrophages in the lesions in vivo and increased lymphocyte proliferation stimulated by myelin antigens and mitogens in vitro [121]. ApoE can bind to sulfatide, a myelin-derived glycolipid, and regulates the sulfatide level either in brain tissue or in CSF [122]. The aggravation of EAE might be a consequence of reduced priming of sulfatide-specific CD1d-restricted regulatory T cells that can inhibit EAE [123].

The aforementioned derived peptide from apoE, COG133, attenuates the severity of EAE by inhibiting production of cytokines and free radicals, as well as by reducing T cell proliferation [124]. COG133 downregulates the activation of microglia and macrophages, and reduces release of TNF-*α*, IL-6, and NO [125]. In vivo, COG133 can also suppress LPS-induced inflammation in CNS [32].

APOE *ε*4 allele results in early cognitive deficits in EAE mice, including deficits in spatial learning and recalling. Regional decreases in choline acetyltransferase in the hippocampus might explain these deficits [126]. This is in accordance with MS studies on human [114, 127]. 

As argued lately, EAE would only be useful as a model of CNS demyelination such as acute disseminated encephalomyelitis, whilst the misleading role of EAE should not be neglected [128]. The isoform-specific effects of apoE on EAE must be properly interpreted now that EAE cannot mimic clinical MS to a fractional.

## 5. Conclusions and Prospectives

The immuno-modulatory functions of apoE have been extensively studied, while only ambiguous or even controversial results have been obtained in elucidating the isotype-dependent effects of apoE on MS and EAE. This is partly due to the difficulty of interpretation of results from animal experiments to a general conclusion. For example, the difference of serum and tissue apoE levels in APOE *ε*2, *ε*3, and *ε*4 Tg mice might be a confounding factor when isoform-dependent effects are studied. Domain interaction (Table 1) distinguishes apoE4 from apoE2 and apoE3 in biological functions, and contributes to the detrimental effects of apoE4 [129, 130]. Because of domain interaction, apoE4 binds preferentially to very low-density lipoproteins, which are more rapidly removed from plasma than other lipoproteins such as HDL, to which apoE3 and apoE2 bind preferentially, resulting a lower level of apoE in serum [130, 131]. The lower level of apoE4 causally depresses the protective role of apoE in both inflammatory and immune responses. Additionally, lipid-free apoE only binds to LRP [132, 133]. Therefore, in interpreting the effects of apoE on immune responses, its lipidation state, concentration, and location of action must be taken into account. The comparison of APOE 4*ε* carriers, especially APOE 4*ε* heterozygotes in MS, with healthy subjects might not be efficient enough to exclude the influence of other APOE *ε* alleles, for no evidence as yet has confirmed a similar or contrary effect among apoE isoforms on susceptibility to or clinical severity of MS. Moreover, interaction between APOE and other genes should be taken into account in studying the pathogenesis of MS, as in other apoE related diseases such as Alzheimer's disease [134]. Anyway, the elucidation of the exact mechanisms by which apoE functions on the immune responses is appealing in that it may provide new insights to the preventive or therapeutic strategies in coping with autoimmune diseases and even other diseases.

## Figures and Tables

**Table 1:** The main differences among apoE isoforms in humans.

Isoform	AA residues		Domain interaction	Binding to LDLR
	112	158		
ApoE 2	Cysteine	Cysteine	No	Low affinity
ApoE 3	Cysteine	Arginine	No	High affinity
ApoE 4	Arginine	Arginine	Yes	High affinity

AA:amino-acid.

LDLR:low-density lipoprotein receptor.

**Table 2:** ApoE genotype frequencies.

Phenotype	Prevalence (%)	
	Utermann et al. [62]	Menzel et al. [63]
E4/4	2.8	2.3
E3/3	59.8	62.7
E2/2	1.0	0.8
E4/3	22.9	20.3
E4/2	1.5	3.0
E3/2	12	11.0

Total subjects	1031	1000
